# Effects of acute creatine supplementation on iron homeostasis and uric acid-based antioxidant capacity of plasma after wingate test

**DOI:** 10.1186/1550-2783-9-25

**Published:** 2012-06-12

**Authors:** Marcelo P Barros, Douglas Ganini, Leandro Lorenço-Lima, Chrislaine O Soares, Benedito Pereira, Etelvino JH Bechara, Leonardo R Silveira, Rui Curi, Tacito P Souza-Junior

**Affiliations:** 1 Postgraduate program in Human Movement Sciences, Institute of Physical Activity and Sports Sciences (ICAFE), Cruzeiro do Sul University, 01506-000, Sao Paulo, SP, Brazil; 2 Department of Biochemistry, University of Sao Paulo (USP-SP), 05508-900, Sao Paulo, SP, Brazil; 3 School of Physical Education and Sports, University of Sao Paulo (USP-SP), 05508-900, Sao Paulo, SP, Brazil; 4 Federal University of Sao Paulo (UNIFESP), campus Diadema, 09972-270, Diadema, SP, Brazil; 5 School of Physical Education and Sports, University of Sao Paulo (USP-RP), 05508-900, Ribeirao Preto, SP, Brazil; 6 Department of Physiology and Biophysics, Biomedical Building I, University of Sao Paulo (USP-SP), 05508-900, Sao Paulo, SP, Brazil; 7 Department of Physical Education, Federal University of Parana, 80215-370, Curitiba, PR, Brazil

**Keywords:** Creatine, Iron homeostasis, Antioxidant, Plasma, Wingate, Anaerobic exercise, Oxidative stress, Uric acid

## Abstract

**Background:**

Dietary creatine has been largely used as an ergogenic aid to improve strength and athletic performance, especially in short-term and high energy-demanding anaerobic exercise. Recent findings have also suggested a possible antioxidant role for creatine in muscle tissues during exercise. Here we evaluate the effects of a 1-week regimen of 20 g/day creatine supplementation on the plasma antioxidant capacity, free and heme iron content, and uric acid and lipid peroxidation levels of young subjects (23.1 ± 5.8 years old) immediately before and 5 and 60 min after the exhaustive Wingate test.

**Results:**

Maximum anaerobic power was improved by acute creatine supplementation (10.5 %), but it was accompanied by a 2.4-fold increase in pro-oxidant free iron ions in the plasma. However, potential iron-driven oxidative insult was adequately counterbalanced by proportional increases in antioxidant ferric-reducing activity in plasma (FRAP), leading to unaltered lipid peroxidation levels. Interestingly, the FRAP index, found to be highly dependent on uric acid levels in the placebo group, also had an additional contribution from other circulating metabolites in creatine-fed subjects.

**Conclusions:**

Our data suggest that acute creatine supplementation improved the anaerobic performance of athletes and limited short-term oxidative insults, since creatine-induced iron overload was efficiently circumvented by acquired FRAP capacity attributed to: overproduction of uric acid in energy-depleted muscles (as an end-product of purine metabolism and a powerful iron chelating agent) and inherent antioxidant activity of creatine.

## Background

Creatine is a glycine-arginine metabolite synthesized in the liver, pancreas, and kidneys and is naturally stored by skeletal and cardiac muscles as an energy supplier in the phosphocreatine form [1]. Muscle phosphocreatine plays a key role in anaerobic ATP production in muscles via the highly exergonic reaction catalyzed by creatine kinase. Thus, creatine monohydrate has become an increasingly popular dietary supplement, particularly for improvement of explosive strength performances [2,3]. Recent findings have also proposed that creatine supplementation could efficiently restrain oxidative processes *in vitro*[4,5]. At least two antioxidant mechanisms are currently suggested for creatine: (i) direct scavenging of hydroxyl (HO^·^) and nitrogen dioxide (NO_2_^·^) radicals [6-8] by the creatine *N*-methylguanidino moiety; and (ii) lasting use of anaerobic energy-supplying pathways because of accumulated creatine and preserved glycogen in skeletal muscles [9-11].

A plethora of data has revealed that reactive oxygen species (ROS) are overproduced during and after anaerobic/resistance exercise, but from cellular sources other than mitochondria [12,13]. Induced by an apparent ischemia-reperfusion process during intense contractile activity of the resistance exercise, accumulating concentrations of AMP in exhausting muscle fibers activate the capillary enzyme xanthine oxidase – belonging to the purine catabolic pathway – which catalyzes the conversion of hypoxanthine into uric acid with concomitant overproduction of superoxide radicals (O_2_^·-^) and hydrogen peroxide (H_2_O_2_) [14]. In turn, O_2_^·-^ and H_2_O_2_ are closely related to the production of the highly reactive hydroxyl radical (HO^·^) by iron-catalyzed reactions (Eqs. 1 and 2) that harmfully initiate oxidizing processes in cells, such as lipoperoxidation [15].

(1) $${\text{Fe}}^{3+}{}_{\left(\text{aq}.\right)}+{\text{O}}_{2}{{}^{\cdot -}}_{\left(\text{aq}.\right)}\to {\text{Fe}}^{2+}{}_{\left(\text{aq}.\right)}+{\text{O}}_{2\;(\text{g})}$$

(2) $${\text{Fe}}^{2+}{}_{\left(\text{aq}.\right)}+{\text{H}}_{2}{\text{O}}_{2(\text{aq}.)}\to {\text{Fe}}^{3+}{}_{\left(\text{aq}.\right)}+{\text{OH}}^{-}{}_{\left(\text{aq}.\right)}+{\text{HO}}^{\cdot }{}_{\left(\text{aq}\right)}$$

Although some information linking iron metabolism and oxidative stress in exercise/sports is currently available, data reporting changes in iron homeostasis of plasma during/after one single bout of exercise compared to antioxidant responses are still scarce. Sources of iron overload in plasma during/after exercise are also unclear. Noteworthy, many authors have reported evidence of a “sport anemia” syndrome in athletes and experimental animals – especially in females – as a result of chronic iron deficiency imposed by prolonged training periods [16,17]. Thus, based on iron-redox chemistry, progressive ROS overproduction could be triggered by iron overload in plasma and extracellular fluids during/after anaerobic exercise [18,19]. Together, these redox changes have been increasingly associated to lower athletic performance, early fatigue, inflammatory processes, and higher risks of post-exercise injuries [20-22].

Thus, this work aims to study the influence of acute creatine supplementation on anaerobic performance and redox status in the plasma of physically active young subjects submitted to the exhaustive Wingate test. Because iron homeostasis is a key factor in triggering oxidative stress, our study monitored total and heme iron release in plasma, ferric-reducing capacity in plasma (FRAP assay), and uric acid and lipid oxidation in plasma immediately before as well as 5 and 60 min after the Wingate test. The novelty of the study relies on the selected redox parameters, which refer to pivotal checkpoints of redox imbalances provoked by the anaerobic exercise.

## Materials and methods

### Standards and reagents

Folin-Ciocalteau reagent, bovine serum albumin (BSA), sodium potassium tartarate, butylated hydroxytoluene (BHT), thiobarbituric acid (TBA), ethylenediamine tetraacetic acid (EDTA) and Triton X-100 were purchased from Sigma–Aldrich (St. Louis, MO, USA). Solvents for chromatography analysis were purchased from Merck (Düsseldorf, Germany). Copper (II) sulphate pentahydrated was obtained from Vetec Química Fina Ltda (Rio de Janeiro, Brazil). All the reagents were of analytical grade and the stock solutions and buffers prepared with Milli-Q (Millipore) purified water. Biochemical kits for plasma/serum heminic-‘free’ iron determinations were purchased from Doles Reagentes e Equipamentos para Laboratórios Ltda (Goiania, Brazil). The kit for uric acid determination was from BioClin Quibasa Ltda (Belo Horizonte, Brazil).

### Subjects

Sixteen male subjects undergraduation students (age, 23.1 ± 5.8 years; height, 175.4 ± 2.3 cm; weight, 81.1 ± 9.3 kg), were invited to participate in the study. All subjects were experienced in cycling activity and were physically active for the last 6 months before the study (at least three times a week). Subjects were randomly split into two groups: placebo- or creatine-supplemented groups. The exercise protocol and all other experimental procedures were approved by the Ethics Committee of School of Physical Education and Sport, University of Sao Paulo, which conforms with the Standards for Research Using Human Subjects, Resolution 196/96 of the USA National Health Council of 10/10/1996 and all consented in writing to the achievement of experimental procedures (physical effort undertaken, sample collection, etc.). The subjects participating in this work attested no use of drugs prohibited by the International Olympic Committee (IOC). In addition, all subjects were not under any systemic or topical medical treatment/therapy for, at least, 60 days before the Wingate test (not even using anti-inflammatory drugs), and had no history of smoking, alcohol use, obesity or systemic disease.

### Creatine supplementation

Creatine group subjects were supplemented five times/day with 4 g creatine monohydrate for a total dosage of 20 g creatine/day for 1 week (dissolved in 500 mL of drinking water). Placebo subjects followed the same supplementation protocol but with 4 g maltodextrin/dose (double-blind study). After 7 days, five athletes (three from the creatine group and two from the placebo group) reported slight gastrointestinal discomfort and decided to leave the study.

### Wingate protocol

The Wingate Test [23] was performed using a leg ergometer (Cybex cycle ergometer; Model Metabolic Systems; Division of Lumex, Ronkonkoma, NY, USA) at the Center for Studies in Exercise Physiology (CEFE) at the Federal University of São Paulo (UNIFESP). In this study, increasing loads up to 10 % of body weight were thoroughly used for male athletes. Volunteers performed a warm-up set of 5 min in the cycle ergometer (25 W) with three sprints of 6 s every minute, followed by a 2-min break before the test. This familiarization test is important to avoid artifacts during the second Wingate test (after supplementation). Each trial was strongly encouraged by the evaluator to achieve maximum possible effort, without raising the trunk from the bicycle seat during the test. After each set of maximal effort, the workload was adjusted to accommodate an active recovery mode (no resistance, 80 rpm, for 3 min). Volunteers were instructed not to perform vigorous physical activity and to avoid drinking caffeinated substances (coffee, chocolate, mate, guarana, energy drinks, and cola) or alcohol within 24 h prior to the tests.

### Blood sampling and plasma preparation

Blood samples (5 mL) were withdrawn from the forearm cubital vein of the volunteers immediately before (t0), as well as 5 min (t5) and 60 min after (t60) the Wingate test, using EDTA-containing Vacutainer kits. Samples were stored in a freezer at −80 °C until analysis. All materials used for blood collection (including syringes, needles, and bottles) were disposable and handled by medical professionals of the CEFE/UNIFESP to prevent potential physical complications.

### Iron content in plasma

Iron concentration in plasma was assayed with a specific biochemical kit from Doles-Bioquímica Clínica (Brazil), using the method first described by Goodwin et al [24]. Currently the method is based on the ferrozine detection (at 560 nm) of ferrous ion released from plasma transferrin by the reducing agent Ferrozine®, which contains: 0.36 M hydroxylamine chloride, 0.10 M glycine, 14 mM thiosemicarbazide, and 0.50 mM octylphenoxypolyetoxyethanol, at pH 2.2 [25]. Total iron released in plasma was calculated by determining the area under curves within the time-span of t0 and t60 (AUC_t0-t60_).

### Ferric-reducing activity in plasma (FRAP)

The ferric-reducing activity in plasma (FRAP) assay was performed as previously described by Benzie & Strain [26] but replacing the iron (II) chelating agent 2,4,6-tripyridyl-*S*-triazine (TPTZ) by its analog 2,3-*bis*(2-pyridyl)-pyrazine (DPP) [27]. Control analytical assays with standard ferrous and ferric ions [Fe(II) and Fe(III), respectively] revealed accurate stoichiometric equivalence between the two chelating agents (data not shown). Briefly, the reactant mixture for FRAP assay contains 10 mM DPP (stock solution prepared in 40 mM HCl) and 20 mM FeCl_3_ in 0.30 M acetate buffer (pH 3.6). To 300 μL of FRAP reactant mixture, 10–20 μL sample is added together with 40–30 μL distilled water (total volume, 350 μL). Absorbance at 593 nm was recorded for 4 min in a microplate reader TECAN (Salzburg, Austria) to determine the rate of Fe(II)-DPP complex formation as compared to a Fe(II) standard curve. Total FRAP was calculated by determining the area under curves within the time-span of t0 and t60 (AUC_t0-t60_).

### Total heme-iron content in plasma

Heme-iron content in plasma was assayed with a specific biochemical kit from Doles-Bioquímica Clínica (Brazil). The method is based on the heme-iron oxidation by the ferricyanide anion contained in a solution with 0.10 M potassium dihydrogenophosphate, 60 mM K_3_[Fe(CN)_6_, 77 mM KCN and 82 mM Triton X-100). Total heme-iron cyanide – which includes heme groups from hemoglobin, myoglobin, and other hemeproteins – is stoichiometrically detected at 540 nm [28,29]. Total heme-iron released in plasma was calculated by determining the area under curves within the time-span of t0 and t60 (AUC_t0-t60_).

### Uric acid determination

Plasma uric acid content was assayed with a biochemical kit from BioClin-Quibasa (Belo Horizonte, Brazil). In the assay mixture, H_2_O_2_ produced from uric acid in the presence of uricase (to form allantoin) is coupled with *p*-hydroxybenzoate and 4-aminoantipyrine oxidation catalyzed by peroxidase to form a pinkish chromophore detected at λ = 505 nm [30]. Total uric acid released in plasma was calculated by determining the area under curves within the time-span of t0 and t60 (AUC_t0-t60_). Furthermore, total uric acid released in plasma within the t0 – t60 interval was correlated with total FRAP, but comparison was purposely made with following groups: (i) subject samples not affected by creatine: pre-placebo, post-placebo, and pre-creatine together; and (ii) post-creatine samples.

### Lipid peroxidation measurements

One of the most frequently evaluated byproducts from lipid peroxidation is malondialdehyde (MDA), which was accurately analyzed here by chromatographic HPLC technique [31]. The biomarker MDA was first dispersed from cellular compartments in a mixture of 50 μL of plasma with 250 μL methanol 30 % for 15 min (4^o^ C) in an ultrasound bath. Afterwards, 100 μL of 0.50 % butylated hydroxytoluene (BHT) were added to the samples to avoid oxidation reactions in the following steps. Molecules of MDA were then converted into a pinkish chromophore by derivatization with 600 μL of a 0.4 % thiobarbituric acid solution (TBA, in 0.20 M HCl) for 30 min at 95^o^ C, under constant mixing. The sample was then filtered (MilliPore nylon membranes, 0.45 μm pore size, 13 mm diameter) and injected (20 μL) in a Shimadzu SCL10A HPLC system provided with LC10AD pumps and fluorescence (RF-10AXL) detector. The MDA-TBA adduct was isocratically eluted by the 65:35 mixture of 25 mM phosphate buffer (pH 6.5) and methanol 30 % through a 0.39 x 30 cm μBondapack C18 column (retention time ~ 6 min) and detected by fluorescence (excitation λ = 515 nm; emission λ = 555 nm). Total areas of MDA peaks of samples were compared with a standard curve obtained with 1,1,2,2-tetraethoxypropane (also in methanol 30 %). Total MDA released in plasma was calculated by determining the area under curves within the time-span of t0 and t60 (AUC_t0-t60_).

### Statistical analysis

All data were analyzed using a 2x2 Factorial (two-way) ANOVA for creatine supplementation and pre-/post variations followed by a post hoc Tukey test to investigate possible interactions between groups (statistical tool VassarStats, on March 7^th^, 2012, available online at: http://faculty.vassar.edu/lowry/anova2u.html). Results were expressed as mean ± SEM of, at least, triplicates of experiments.

## Results

After supplementation but before the anaerobic test (W_post_; section 2.4), creatine-fed subjects showed a significant 2.4-fold increase in plasmatic iron (t*0*_post_/t*0*_pre_; p < 0.005), heme iron (80 %; p < 0.05), and FRAP (3-fold; p < 0.05) compared with t*0*_pre_ scores, while the placebo group showed no significant change (Table 1). These results were interpreted as the subjects’ basal levels because they were obtained from blood samples collected before the exhaustive Wingate test (t*0*_pre_ and t*0*_post_); thus, they were not related to the oxidative stress imposed by anaerobic exercise. On the other hand, two-way ANOVA test followed by post hoc Tukey’s analysis revealed moderate heterogeneity between group placebo and creatine-fed before the exhaustive Wingate test (Table 1) for all redox parameters analysed, except lipid peroxidation (MDA measurements). Nevertheless, all values found in groups before the Wingate test (t*0*_pre_ for both placebo and creatine-fed groups; Table 1) were within the regular range in plasma of human subjects and, thus, could reflect the natural variations expected for human populations. Biochemical changes in the iron-related parameters were observed together with 28 % lower levels of lipid oxidation (t*0*_post_/t*0*_pre_; Pearson’s r < 0.01), whereas the placebo group was unaltered. Conversely, no change in the total uric acid content in plasma was observed in t*0*_post_/t*0*_pre_ ratios from placebo and creatine groups (Table 1). Weight and percent body fat were also unaltered after acute creatine supplementation (data not shown).

**Table 1:** **Redox biomarkers of anaerobic exercise in plasma of subjects before (t*0*_pre_) and after 20 g/day creatine monophosphate supplementation for 1 week (t*0*_post_)**

	**Placebo**		**Creatine**	
	**t*0***_**pre**_^**(a)**^	**t*0***_**post**_^**(b)**^	**t*0***_**pre**_^**(c)**^	**t*0***_**post**_^**(d)**^
Iron content *(μg/dL)*	33.3 ± 7.8 (§c;*d)	26.3 ± 5.5 (*c)	12.2 ± 3.4 (§a;*b,d)	23.7 ± 1.8 (*a,c)
Heme-iron*(mg/mL)*	7.94 ± 0.43(*c)	7.89 ± 0.24 (*c)	4.77 ± 0.93(*a,b,d)	6.47 ± 0.13 (*c)
FRAP *(μmolFe*^*2+*^*/min/mL)*	0.057 ± 0.011(§c,d)	0.077 ± 0.020(§d;*c)	0.110 ± 0.014 (§a,d;*b)	0.300 ± 0.038(§a,b,c)
MDA *(μmol/L)*	0.129 ± 0.023	0.148 ± 0.043	0.186 ± 0.050	0.129 ± 0.025
Uric acid *(mg/mL)*	1.62 ± 0.94 (§c,d)	1.62 ± 0.75 (§c,d)	2.93 ± 0.49 (§a,b)	3.44 ± 0.39 (§a,b)

(§) p < 0.005; (#) p < 0.01; (*) p < 0.05.

Regarding performance in the Wingate test (Table 2), neither anaerobic capacity (AnC; p = 0.1275) nor total workload (TotalWL; p = 0.1040) were significantly altered by creatine supplementation, whereas maximum anaerobic power was significantly increased by 10.5 % (AnP_peak_; p = 0.0029) and the fatigue index showed a strong trend for anaerobic effort reduction upon creatine supplementation (p = 0.0890). The fatigue index was not determined in the placebo group. Discrepancies between W_pre_ of placebo and creatine (basal values in Table 2) were identified herewith by the two-way ANOVA test, but we assumed that such heterogeneity would not represent a relevant factor in explaining major changes in redox/metabolic parameters or anaerobic performance indexes.

**Table 2:** **Indexes of anaerobic performance of subjects during a Wingate protocol before (W**_**pre**_**) and after (W**_**post**_**) 20 g/day creatine monophosphate supplementation for 1 week (double-blind study; MEAN ± SEM)**

	**Placebo**		**Creatine**	
	**W**_**pre**_^**(a)**^	**W**_**post**_^**(b)**^	**W**_**pre**_^**(c)**^	**W**_**post**_^**(d)**^
AnP_peak_*(W/kg)*	9.68 ± 1.08 (*c,d)	10.33 ± 0.80 (*d)	11.4 ± 0.5 (*a,d)	12.6 ± 0.6 (*a,b,c)
AnC *(W/kg)*	5.05 ± 0.52 (#c,d)	5.08 ± 0.35 (#c,d)	8.1 ± 0.4 (#a,b)	8.5 ± 0.8 (#a,b)
TotalWL *(J/kg)*	151.8 ± 15.8 (#c,d)	152.3 ± 10.5 (#c,d)	241.1 ± 12.4(#a,b)	255.0 ± 21.2(#a,b)
Fatigue index *(%)*	n.d.	n.d.	60 ± 8	40 ± 8

(§) p < 0.005; (#) p < 0.01; (*) p < 0.05.

n.d. = not determined.

**Figure 1:**
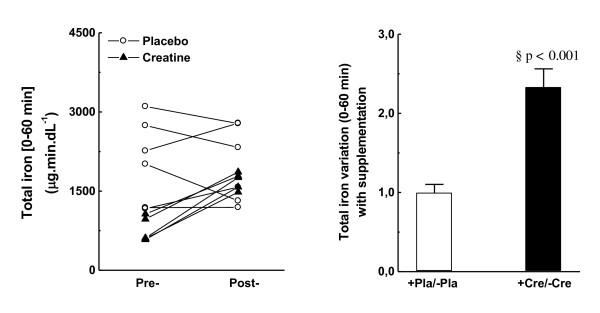
**Total iron released in plasma from t0 (immediately before the Wingate test) until t60 (60 min after).** (A) Individual pre-/post-variation with placebo or creatine supplementation; (B) Average pre-/post-variation with placebo or creatine supplementation.

Total releases of iron, heme iron, FRAP, MDA, and uric acid plasma by the Wingate test were calculated from the AUC within t*0* and t*60* and were compared as pre- and post-placebo *versus* pre- and post-creatine scores. Figure 1A shows the pre/post variation of total iron released within the t0–t60 interval in both placebo and creatine groups. All creatine-fed subjects demonstrated higher loads of released iron with exercise after supplementation (2.4-fold higher; p < 0.001), whereas the placebo did not vary (Figure 1B). Noteworthy, the heterogeneity of basal iron content in plasma of placebo- and creatine-fed subjects was also reflected in observed discrepancies between groups when evaluating total iron content in plasma within the t0-t60 interval (Pearson’s r < 0.05, not shown in Figure 1A).

**Figure 2:**
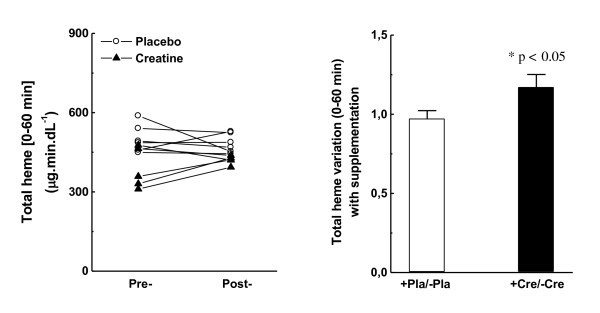
**Total heme-iron released in plasma from t0 (immediately before the Wingate test) until t60 (60 min after).** (A) Individual pre-/post-variation with placebo or creatine supplementation; (B) Average pre-/post-variation with placebo or creatine supplementation.

Total released heme iron in the creatine group did not increase as abruptly as the total iron content, but the post/pre variation was still significantly higher (17 %; p < 0.05; Figure 2A and B). The placebo group was unaltered regarding post/pre variation.

**Figure 3:**
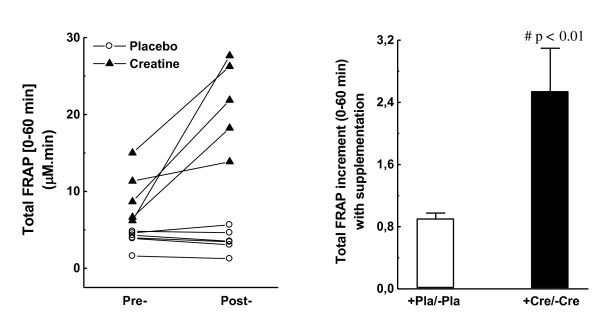
**Ferric-reducing activity in plasma (FRAP) from t0 (immediately before the Wingate test) until t60 (60 min after).** (A) Individual pre-/post-variation with placebo or creatine supplementation; (B) Average pre-/post-variation with placebo or creatine supplementation.

As an antioxidant response against the observed massive amount of iron ions released during (Wingate) exercise, total FRAP in creatine-fed individuals also increased by almost 3-fold in the post/pre comparison (Figure 3B; p < 0.01). Moreover, the heterogeneity of basal FRAP capacity of placebo- and creatine-fed subjects was reproduced when total FRAP capacity was measured in subjects within the t0-t60 interval (Pearson’s r < 0.05, not shown in Figure 3A). We assumed that none of the basal variations found for iron-related redox parameters could drastically interfere in the proposed antioxidant action of creatine (or one of its metabolites) following the exhaustive Wingate test, since all these values were within the regular range of human populations.

**Figure 4:**
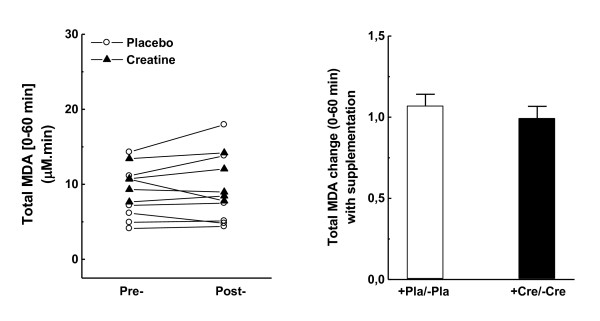
**Malondialdehyde content plasma (MDA) from t0 (immediately before the Wingate test) until t60 (60 min after).** (A) Individual pre-/post-variation with placebo or creatine supplementation; (B) Average pre-/post-variation with placebo or creatine supplementation.

In contrast to the diminished scores observed in t*0* samples of creatine-fed individuals (Table 1), no significant change was observed between placebo and creatine groups regarding the total MDA released in plasma within the t0–t60 interval (Figure 4A-B).

**Figure 5:**
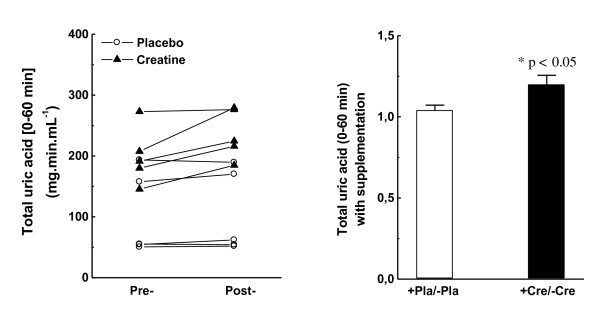
**Uric acid content plasma (MDA) from t0 (immediately before the Wingate test) until t60 (60 min after).** (A) Individual pre-/post-variation with placebo or creatine supplementation; (B) Average pre-/post-variation with placebo or creatine supplementation.

Finally, acute creatine supplementation resulted in a significant post/pre increase of 20 % (p < 0.05) in the uric acid released in plasma within the t0–t60 interval, whereas the placebo group did not vary significantly (Figure 5A and B).

**Figure 6:**
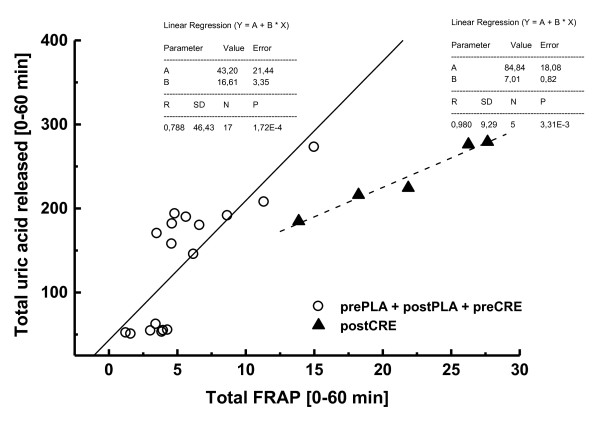
**Linear regression (and equations in insets) between total uric acid content and ferric-reducing activity in plasma (FRAP) of placebo and creatine-fed subjects within 60 min of the exhaustive Wingate test.** Samples collected in subjects after creatine supplementation (postCRE) were compared to samples collected from placebo group (both before and after supplementation, prePLA, and postPLA, respectively) and from subjects before creatine supplementation (preCRE).

Interestingly, the total uric acid released in plasma within the t*0*–t*60* interval (Wingate test) was very well correlated with the total FRAP released, both in subjects supplemented with creatine (R = 0.980; black triangles; Figure 6) or without creatine (R = 0.788, here purposely grouped as pre-placebo, post-placebo, and pre-creatine; open circles; Figure 6). However, upon creatine supplementation (post-creatine samples), FRAP increase is less dependent on total uric acid than in samples that lack the creatine effect (namely pre-placebo, post-placebo, and pre-creatine samples). Linear regression equations for post-creatine and grouped pre-placebo, post-placebo, and pre-creatine samples were as follows, respectively: (i) *(Total Uric Acid) = 84.8 + 7.01(Total FRAP), R = 0.980*; and (ii) *(Total Uric Acid) = 43.2 + 16.61(Total FRAP); R = 0.788* (insets, Figure 6).

## Discussion

Creatine has long been credited as an efficient ergogenic supplement that improves the anaerobic power of athletes submitted to high-intensity, short-duration tests [1,3]. The metabolic strategy is supported by the previous creatine overload in muscle fibers (particularly type-II) and enhancement of ATP generation for extra power output during early/anaerobic stages of exercise. The maximum anaerobic power was significantly increased by 10.5 % after acute 20 g/day creatine supplementation (Table 2), together with strong tendencies for increased total workload and reduced fatigue index, although not significant in the present study. However, creatine has also been shown to have a role as an antioxidant compound that hampers overproduction of harmful reactive oxygen species (ROS) within contractile skeletal muscles during exercise [6,32]. This hypothesis is in line with recent findings by Sestili et al. [33] who demonstrated that creatine treatment can directly prevent cell death in C2C12 myoblasts due to its antioxidant activity. Regarding mechanisms, due to its substantial absorption and dose-dependent accumulation in plasma following supplementation [34], creatine is supposed to exert a direct scavenging effect against ROS produced in plasma – with concomitant minor chelating action [7] – that enhanced blood antioxidant capacity in creatine-fed subjects (FRAP, Table 1). Neither creatine itself nor any of its metabolites (e.g. creatinine) were directly measured here. Therefore, we cannot exclude the hypothesis of a co-adjutant chelating role of one of the creatine metabolites in plasma following its acute supplementation. Further studies are necessary to better address this hypothesis.

Iron ions are reportedly released in plasma during/after strenuous exercise, but intracellular or plasmatic sources are still relatively obscure [18,19]. Regarding total iron released in plasma (AUC_t*0*-t*60*_), creatine supplementation resulted in higher amounts released during/after 60 min of the exhaustive Wingate test (2.4-fold higher; Figure 1A and B). However, the same 2.4-fold higher iron content was also observed in creatine-fed subjects at rest, with lower increment from heme-iron (t*0*_post_/t*0*_pre_, Table 1). Thus, it is tempting to suggest that the pre-acquired increased iron content in plasma during the creatine supplementation period was responsible for a similar increase during/after exercise, indicating that no other source was mainly contributing to the total iron load in plasma during exercise. Accordingly, heme iron clearly provided a minor contribution (20 %) to the observed massive iron release in plasma during/after exhaustive exercise in creatine-fed subjects tested in the present study (Figure 2A-B). Based on the presented data, hemolysis and rhabdomyolysis are processes possibly less related to iron release in the plasma of placebo subjects during/after Wingate test. These data are in agreement with new findings that suggest ferritin and, perhaps, transferrin are the major free iron sources that trigger oxidative stress during exercise [35]. Notably, free iron actually refers to metal ions bound to low-molecular-weight metabolites in biological fluids (such as ascorbate, adenosine, and citrate) that can still catalyze the Fenton-reaction [36], a natural chemical process that produces one of the most aggressive ROS, the hydroxyl radical (HO^·^). Early studies have shown that alterations in the extent of iron storage in tissue ferritins (rat liver and spleen) in vivo coincide with experimentally induced alterations in oxidative metabolism within cells: e.g. aerobic conditions (or experimental procedures) leading to ATP synthesis will favor the movement of serum iron to liver and spleen ferritins, whereas tissue hypoxia leading to ATP degradation will favor the release of ferritin iron to the serum and will inhibit the movement of serum iron to tissue ferritin [37]. Despite of that, none of these experimental conditions included strenuous aerobic or anaerobic exercises. Furthermore, in vitro assays demonstrated that the xanthine oxidase system plays an important role in the process of iron reduction (ferric to ferrous ions) and release from hepatic ferritin in hemorrhagic shock animals [38].

Vigorous contractions during high-energy-demanding anaerobic exercises activate O_2_-consuming xanthine oxidase (XO) at local vascular endothelium [39]. In exhausting fast-twitch fibers (when ATP supply is limited), accumulation and subsequent deamination of AMP enhance inosine conversion to hypoxanthine. Under these circumstances, accumulated hypoxanthine is efficiently oxidized by pre-activated XO to xanthine, and ultimately to uric acid, which also renders high production of O_2_^·-^, H_2_O_2_, and other ROS [40,41]. Thus, uric acid content in plasma is related to intracellular energy balances in muscle fibers, and thus performance, because the degree of adenine catabolism is regulated by [ATP]:[AMP] ratios [42]. Accordingly, subjects supplemented with creatine showed approximately 20 % higher total uric acid released in plasma than the placebo group (Figure 5A and B), which is also slightly related to the 10.5 % higher scores of maximum anaerobic performance (Table 2). Xanthine oxidase-based ROS overproduction could culminate in harsh oxidative insult to muscle fibers, unless efficient antioxidant systems are promptly activated. This condition is particularly enhanced by the massive release of Fenton-catalytic iron metals during/after exhaustive exercise [18,19]. Independently, with a subcellular or extracellular source of iron overload, all individuals displayed adequate plasma antioxidant responses as suggested by the observed proportional increases in ferric-reducing activities in plasma during/after exercise (FRAP; Figure 3A and B) and by unaltered levels of lipid oxidation until 60 min after the Wingate test (Figure 4A-B). Moreover, the iron content in plasma and FRAP were also augmented in creatine-supplemented subjects at rest (t*0*_post_/t*0*_pre_ in Table 1), whereas 28 % lower levels of lipid oxidation were also found in plasma (Table 1). Taken together, these facts corroborate the hypothesis of how tightly iron homeostasis is controlled in animals and humans possibly to prevent metal-catalyzed formation of aggressive ROS such as HO^·^ radical.

Uric acid, the final product of purine catabolism, has been proven to be an efficient antioxidant and chelating agent for iron ions [43]. Furthermore, uric acid alters the redox potential of chelated Fe^2+/3+^ and, thus, seems to act as an antioxidant by preventing Fenton-like reactions in many biological systems and the oxidation of other antioxidant systems, such as ascorbate [36]. Interestingly, addition of uric acid to the culture media (at nonphysiological concentrations) limited polyunsaturated fatty acid oxidation in the erythrocyte membrane and prevented hemolysis *in vitro*[44] similarly as proposed in the present study by the observed lower heme iron release (Figure 2A-B). As the missing part of the puzzle, uric acid is apparently one of the major contributors for the FRAP antioxidant response during/after anaerobic exercise, as strong linear correlations between FRAP and uric acid have already been reported in pentathlon competition horses (Pearson’s r = 0.78) [45,46]. This concept is fully consistent with our data presented in Figure 6.

With regard to total amounts released in plasma during/after the Wingate test (UAC_t*0*-t*60*_), uric acid and FRAP were very well correlated in both placebo and creatine groups (Figure 6). However, notably, higher FRAP scores found in creatine-fed subjects is less dependent on total uric acid than in samples that lack the creatine effect (namely pre-placebo, post-placebo and pre-creatine). This suggests that an additional chelating (and Fe^2+/3+^ redox-inactivating) agent is present in the plasma of creatine-fed subjects during/after anaerobic exercise to provide an extra antioxidant role, and the best candidate is creatine itself. Even considering the well-described antioxidant activity of creatine *in vitro* and *in vivo*[6,7], whether such an antioxidant/chelating role is actually performed by creatine, or any of its metabolites (e.g., creatinine), remains unclear and further studies are necessary.

## Conclusions

Our data are consistent with the hypothesis that creatine supplementation rebalances iron homeostasis both at rest and during/after anaerobic exercise. Such a risky condition concerning ROS production and oxidative stress is circumvented by a proportional increase in FRAP provided by uric acid and by creatine itself (or its metabolites) in plasma. Under these circumstances, lipid oxidation scores were unaltered after the exhaustive Wingate test. Although acute supplementation of creatine only resulted in modest improvement of anaerobic capacity (an attempt to minimize adverse renal dysfunctions of its chronic use), it also provided an additional antioxidant protection in plasma of supplemented subjects. Unfortunately, it is not well stated that the improved antioxidant capacity of plasma will result in better anaerobic performance, but general health benefits are truthfully suggested here, for example in restraining post-exercise inflammatory processes. Anaerobic exercise to exhaustion reveals an intricate redox mechanism, which is vigorously orchestrated by iron release and FRAP responses, with uric acid as the main protagonist. Creatine herewith is an uprising actor stealing the scene in our new adaptation of the story.

## Abbreviations

AMP: adenosine monophosphate; ATP: adenosine triphosphate; AnC: anaerobic capacity; AnP: anaerobic power; AUC: area under curve; BHT: butylated hydroxytoluene; BSA: bovine serum albumin; FRAP: ferric-reducing activity of plasma; H2O2: hydrogen peroxide; HO·: hydroxyl radical; MDA: malondialdehyde; O2·-: superoxide radical; ROS: reactive oxygen species; TBA: thiobarbituric acid; TotalWL: total workload; XO: xanthine oxidase.

## Competing interests

The results of the present study do not constitute endorsement of any products by the authors or by ACMS or other organizations. The authors herewith have no competing interests.

## Authors’ contributions

Our study entitled “Effects of acute creatine supplementation on iron homeostasis and uric acid-based antioxidant capacity of plasma after wingate test” is here authored by 09 scientists, namely: Marcelo P. Barros, Douglas Ganini, Leandro Lorenço-Lima, Chrislaine O. Soares, Benedito Pereira, Etelvino J.H. Bechara, Leonardo R. Silveira, Rui Curi and Tácito P. Souza-Junior. We here present their effective contributions to the MS. Dr. Marcelo P. Barros and Dr. Tácito P. Souza-Junior – first and corresponding authors, respectively – are mentors of the study (concept and design) and organizers of the experimental activities and responsible for manuscript preparation. M.Sc. Leandro Lorenço-Lima and Dr. Benedito Pereira were responsible for the supplementation program/procedure and acquisition of anaerobic performance data during the Wingate test. Dr. Douglas Ganini and Chrislaine O. Soares (Ph.D. student) were involved in HPLC analyses for lipid oxidation data. Prof. Etelvino Bechara is their current supervisor and also fully reviewed (observations and comments) our MS in order to improve the quality of our contribution. Finally, Dr. Leonardo R. Silveira and Prof. Rui Curi substantially contributed to the improvement of our physiological approach of our hypothesis. These authors also revised the final version of our manuscript.
